# From homeostasis to behavior: Balanced activity in an exploration of embodied dynamic environmental-neural interaction

**DOI:** 10.1371/journal.pcbi.1005721

**Published:** 2017-08-24

**Authors:** Peter John Hellyer, Claudia Clopath, Angie A. Kehagia, Federico E. Turkheimer, Robert Leech

**Affiliations:** 1 Department of Bioengineering, Imperial College London, London, United Kingdom; 2 Centre for Neuroimaging Sciences, Institute of Psychiatry, Psychology and Neuroscience, King’s College London, London, United Kingdom; 3 Computational, Cognitive and Clinical Neuroimaging Laboratory (C3NL), Imperial College London, Hammersmith Hospital, London, United Kingdom; Ghent University, BELGIUM

## Abstract

In recent years, there have been many computational simulations of spontaneous neural dynamics. Here, we describe a simple model of spontaneous neural dynamics that controls an agent moving in a simple virtual environment. These dynamics generate interesting brain-environment feedback interactions that rapidly destabilize neural and behavioral dynamics demonstrating the need for homeostatic mechanisms. We investigate roles for homeostatic plasticity both locally (local inhibition adjusting to balance excitatory input) as well as more globally (regional “task negative” activity that compensates for “task positive”, sensory input in another region) balancing neural activity and leading to more stable behavior (trajectories through the environment). Our results suggest complementary functional roles for both local and macroscale mechanisms in maintaining neural and behavioral dynamics and a novel functional role for macroscopic “task-negative” patterns of activity (e.g., the default mode network).

## Author summary

In recent years, there has been growing interest in using computational models based on the human structural connectome to better understand the brain. These simulations typically investigate spontaneous neural dynamics, in the absence of tasks, sensory input or motor output. Here, we take a different approach, embodying a computational model of spontaneous neural dynamics to control a simulated agent, with sensory input from and motor output to a simulated environment. Embodying the model radically changes how the model operates and changes how we understand the computational mechanisms. We observe interesting brain-environment feedback interactions and observe how different homeostatic systems are needed to compensate for this feedback. We observe this both in the simulated neural dynamics and the behavior of the embodied agent. These findings suggest novel functional roles for homeostatic systems in maintaining neural dynamics and behavior and for the poorly understood default mode network pattern of activity reported in functional neuroimaging in humans and animals.

## Introduction

In recent years, empirical and theoretical work indicates that homeostatic mechanisms play an important role in the regulation of neural activity. At the microscopic level, the balance of local excitation and inhibition (E/I) has important computational properties [1–2]. The balance of E/I can be maintained in such circuits using relatively simple local homeostatic rules based on inhibitory plasticity (e.g., [3–6]). At a completely different scale, evidence from functional MRI suggests the balance of activity across brain regions may be an important organizing principle in macroscopic neural dynamics; networks of task associated regions typically show increased activity matched by relative de-activation of other ‘task negative’ macroscopic networks (e.g., [7–9]). The default mode network (the classic task negative network) is spatially situated between different networks often activated during externally focused tasks [10]; in our previous work, we suggested that at the whole brain level ‘task negative’ network activity may act to counterbalance task activation in other brain regions, forming a ‘network balance’ analogue of the local computational motifs driven by inhibition seen at smaller scales [11–14].

**Fig 1:**
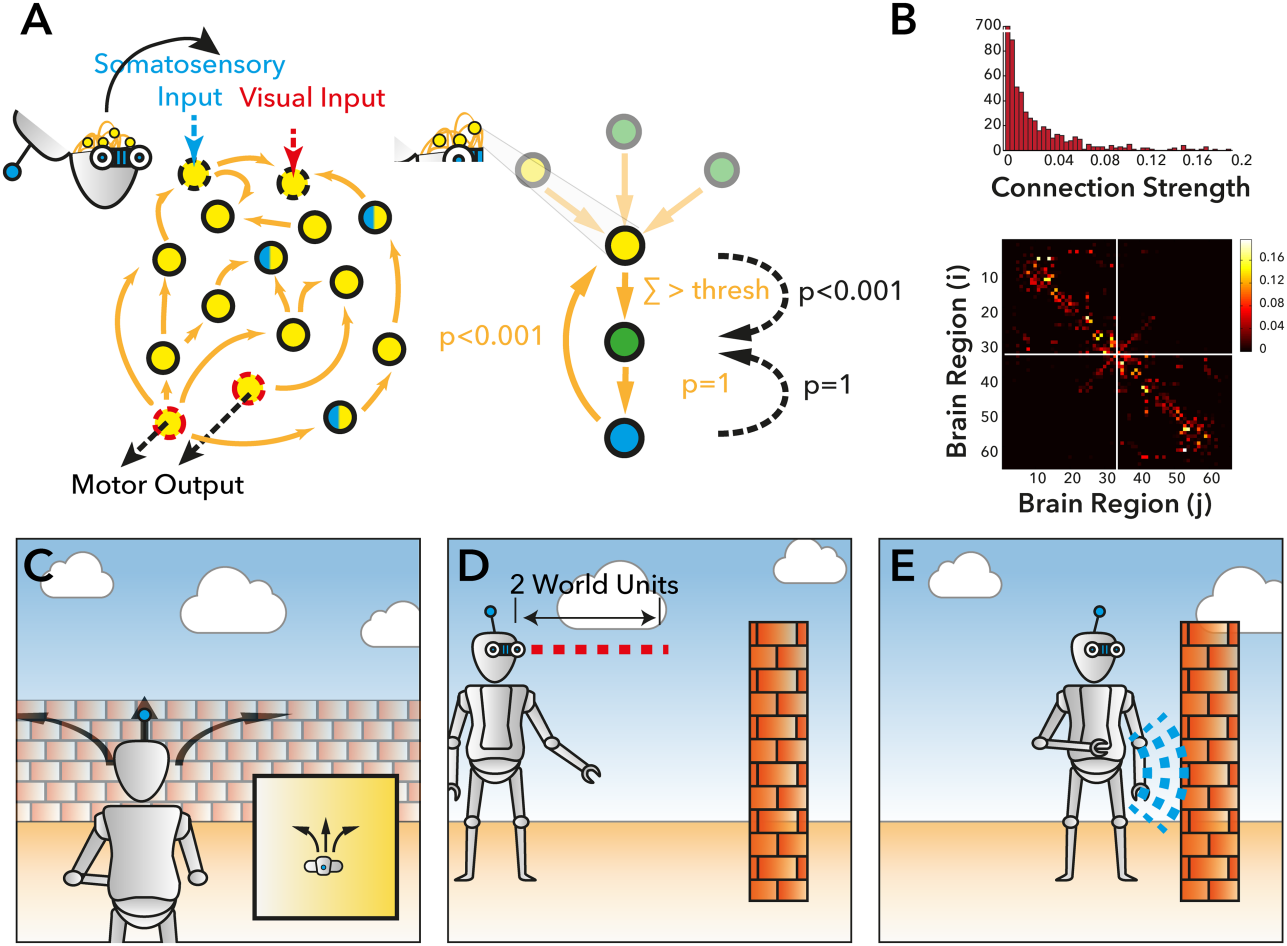
Embodiment of the Greenburgh Hastings model within the agent. **A)** A Schematic overview of the network (Left) and local (Right) dynamics of the Greenburgh-Hastings (GH) model. In our model, specific nodes were defined as ‘somatosensory’, ‘visual’ nodes and motor nodes (see Materials and methods). Using this basic framework, we defined four different forms of the computational model 1) A non-plastic model, where the strength of connectivity and threshold (thresh) between individual nodes (orange arrows) was held consistent over time. 2) a Local homeostatic model, where the threshold in each node could vary according to the level of local activity (see Materials and methods). And 3) a model where nodes (crossed blue and yellow nodes) defined at the macroscopic scale were forced to deactivate to counter-balance activity within somatosensory and visual nodes, and 4) a model which combined the local and macroscopic balance mechanisms of 2+3 **B)** A simple overview of the 66 node DSI connectivity matrix used in the current work to constrain the network dynamics of the GH model. **C-E)** The activity within the motor nodes when the agent was placed into the virtual environment were translated into ‘Forward’ and ‘Turn’ commands **(C)** when the agent was within 2 ‘world units’ of an obstacle **(D)** the ‘visual’ nodes would be automatically set to active. When the Agent collided with an obstacle **(E)**, the ‘somatosensory node’ was set to active.

**Fig 2:**
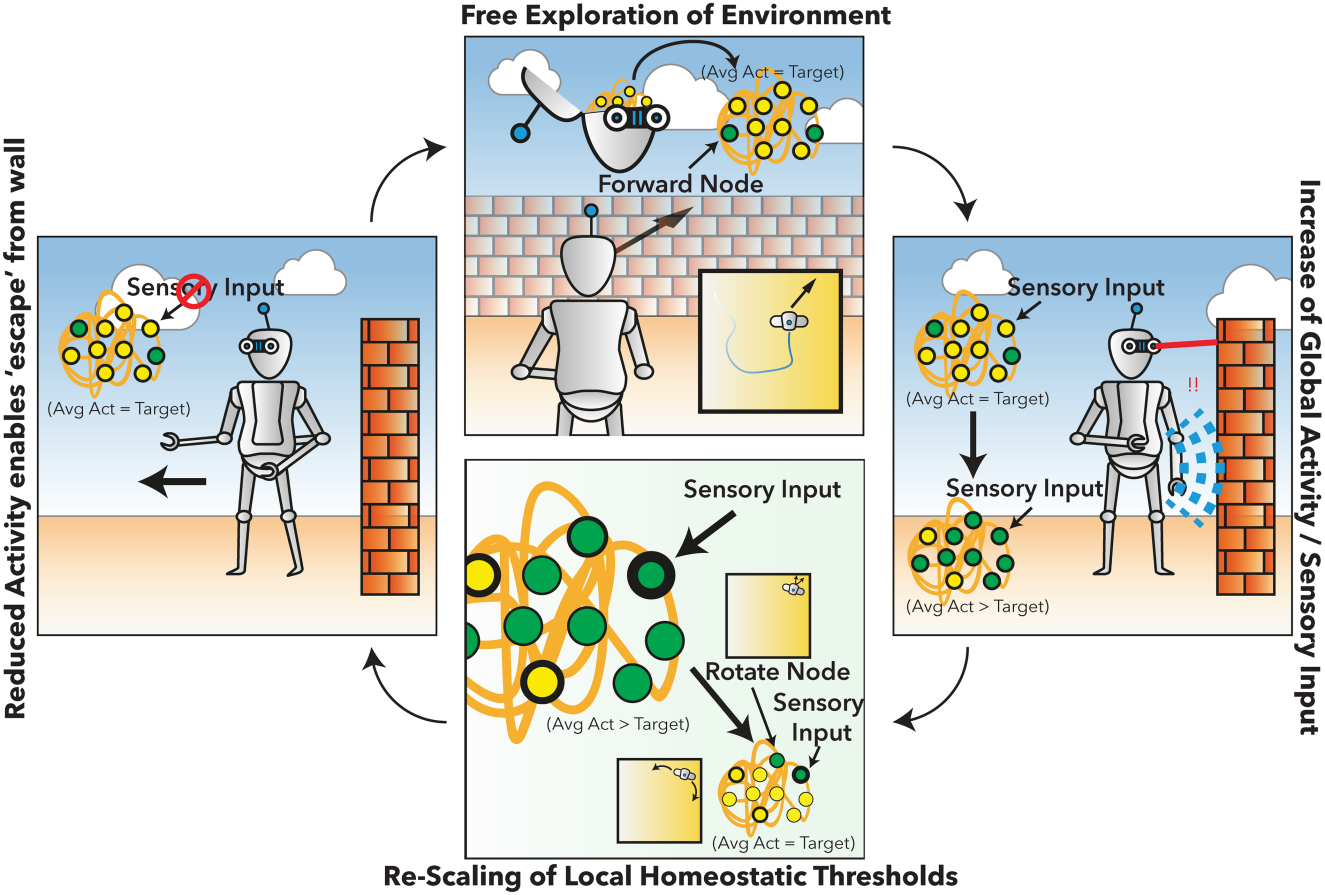
Behavioral interaction of the agent with simulated neural dynamics. **Top)** In the normal state, the agent displays neural dynamics according to the target rate defined in the homeostatic rule of the computational model. **Right)** When the agent interacts with the environment, this causes sensory input to the system, which destabilizes the dynamics of the model leading to an increase in global activity above the target function. **Bottom)** The increase in activity within the neural model leads to a re-calibration of the local threshold values to re-gain the target value. Without this local homeostasis, the model continues to show high dynamics and can ‘stick’ in a wall ‘trap’ due to a consistent activation of the forward and turn nodes. A reduction on overall global activity generated by the homeostatic feedback re-sets the model to the target value, and enables simple movements to emerge such as turning away from the wall. **Left)** Once free of an environmental obstacle, sensory input is reduced, which transiently leads to an undershoot of the model activity relative to the target. This allows the agent to safely navigate away from the obstacle and freely explore the environment.

Computational simulations have suggested the importance of homeostatic mechanisms in regulating neural dynamics; facilitating complex patterns of neural activity (e.g., [15–16]). However, such computational models typically simulate the brain at rest or under highly constrained task settings. In the present work, we explore the regulatory role of homeostatic mechanisms underlying simple behavior. We adapt an established simulation of basic neural dynamics—the Greenberg-Hastings model [17], incorporating information about human structural connectivity [18] (Fig 1A). At rest (i.e., with no environmental embodiment), this model has been shown to approximate empirical functional connectivity patterns observed macroscopically [19]. In the model at rest, dynamics arise from low-probability random firing which propagates through recurrent connections. The large-scale dynamics of the model can be controlled by a single local inhibition parameter at each node, which regulates the propagation of incoming excitatory activity to connected nodes in the network. To explore the interaction between brain and environment, we adapt this simple model by ‘embodying’ it into a simple virtual environment. We begin by defining an agent that can move within a 2-dimensional plane, bounded by surrounding walls (see Fig 1C). Movement of the agent within the virtual environment was determined by activity within two pre-defined “motor” nodes within the computational model. Sensory input to the model was defined by direct manipulation of a group of task-positive nodes (TP) within the model to ‘activate’ in response to “sensory” input defined using a collection of virtual sensors embedded within the agent. Two pairs of bilateral nodes reacted to “visual” input from the environment to the model and one pair of “somatosensory” nodes activated if the agent collided with the bounding walls of the environment. This set-up leads to brain/environment interactions as follows (Fig 2):

1. Sensory input from the environment evokes regionally specific visual and sensory activity within the model.
2. This exogenously evoked neural activity alters neural dynamics as evoked activity propagates through the recurrent network.
3. Altered network dynamics cause alterations in motor output from the model leading to alterations in the agent’s trajectory through the environment which in turn alters subsequent sensory input.

This form of closed-loop interaction introduces non-stationary dynamics; in addition, it can lead to ‘traps’ where the agent moves into an area with large amounts of sensory input or no sensory input and then cannot leave that area. For example, in an area with no sensory input, activity in the model can become pathologically low, and the agent stops movement. Equally, in an area with large amounts of sensory input activity, there can be too much activity, which can lead to pathological motion (e.g., running forward into a wall forever). These types of brain-environment interaction potentially present a challenge for computational modelling approaches which focus on the emergence of spontaneous, rich dynamics only at rest. These models often rely on careful parameterization to remain in a specific dynamic regime, and so typically are investigated in static situations (i.e., where the input to the model is stationary, such as Gaussian noise). In such models, changes to the model input typically lead to destabilization of the dynamics (i.e., a shift to either random, saturated, or absent patterns of activity).

To explore the effect of homeostatic control on the stabilization of neural dynamics in this system, we considered two putative mechanisms of dynamic homeostasis:

1. A variation on the local (i.e., within-node) homeostatic plasticity rule presented in [3] and employed in a similar macroscopic neural model in [20–21]. This mechanism adjusts the local inhibition (i.e., propagation threshold) at each node, balancing against incoming excitatory activity from other nodes, and so driving time-averaged local activity to approximate a pre-specified, small target activity rate.
2. A model of macroscopic balancing such that the activity of the task positive ‘sensory’ computational nodes is balanced across regions (i.e., non-locally) by additional bilateral “task-negative” nodes (TN). The choice of the TN nodes was loosely based on the default mode network pattern of task-evoked relative deactivations from fMRI/PET [22], which we have previously suggested, may constitute a macroscopic balancing system [11].

We explore the extent to which these two balancing systems may perform complementary roles in maintaining flexible dynamics in the case of closed-loop brain-environment interactions. We assess the agent’s neural dynamics and trajectory through the environment, and demonstrate that these balancing mechanisms allow the agent to escape constrained environment-brain feedback loops and more completely traverse the environment.

## Results

### Functional and behavioral dynamics in a model without homeostatic plasticity

**Fig 3:**
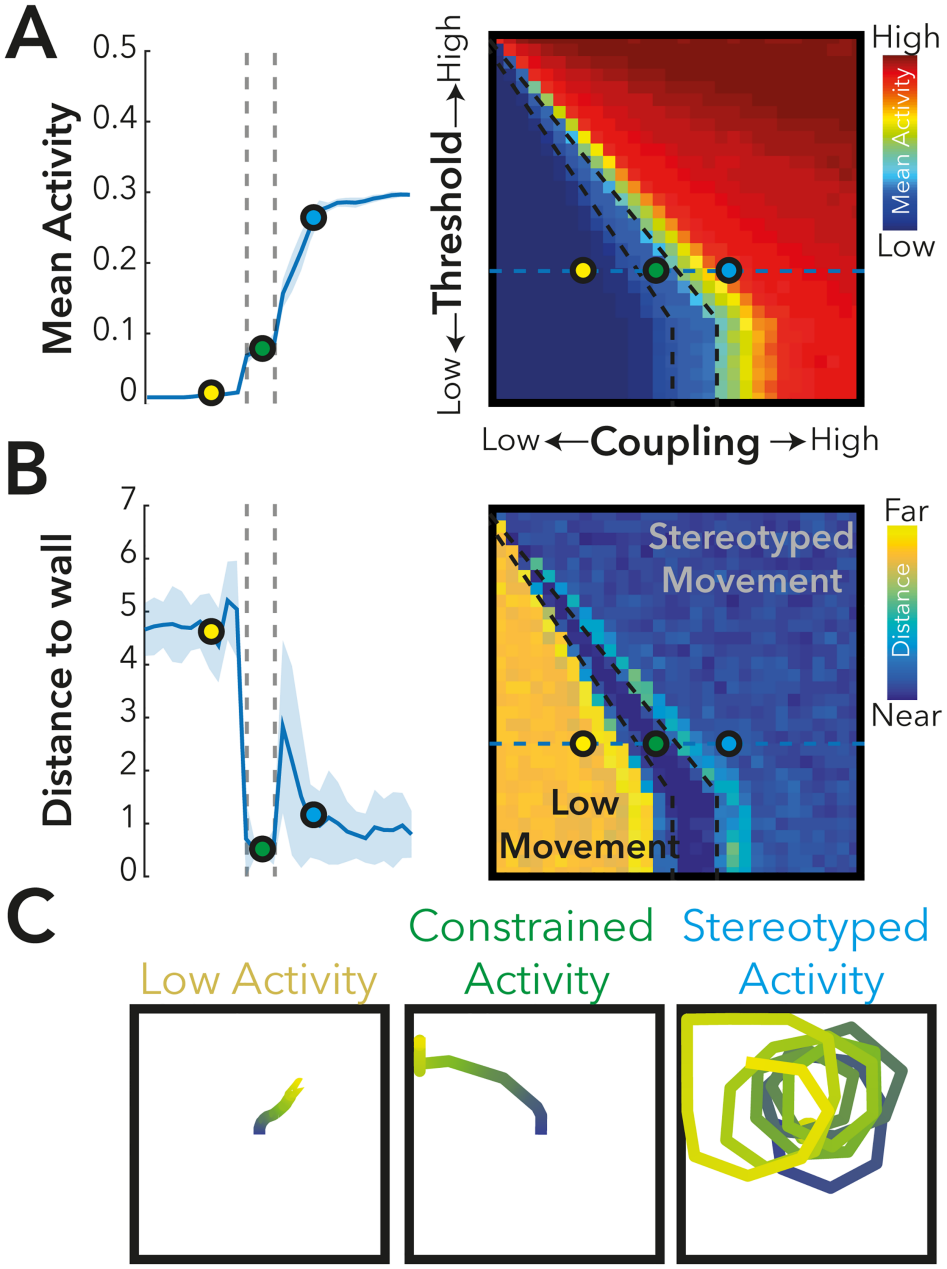
An exploration of the neural and behavioral dynamics of the ‘Non-plastic’ model. Here we explored the effect of altering the coupling (the overall scaling factor of the connectivity matrix) and Threshold, on the dynamics of the computational model, as well as the behavioral characteristics of the Agent. **A)** Mean activity in the model demonstrates a phase transition in terms of the mean activity of the model over time (simulations of 5000 epochs for each parameter pair presented), from a region of low activity (yellow marker), to a region of high activity (blue marker). These simulated dynamics had a behavioral effect on the Agent **(B-C)** where there was either high model activity and highly stereotyped behavior (i.e., running continuously in circles), whilst the low activity model showed little behavioral activity (i.e. movement of the agent). At the phase transition (green marker), the agent moves, but eventually interacts with the boundary of the environment where the model receives increased ‘sensory’ input which leads to increased movement, resulting in the agent becoming ‘stuck’ **(C)**.

In the absence of any homeostatic mechanisms, we explored the extent to which the dynamics of the computational model relate to the motor output of the agent (Fig 3). We started by exploring two key control parameters of the model—the threshold for incoming activity to a node to propagate (Threshold), and the strength of coupling between each region (Coupling). We explored the extent to which both simulated activity and movement of the agent are constrained by these factors.

In the case of low coupling, the model remains in a state of low activity (Fig 3A). As coupling increases the model rapidly transitions through a phase transition to a ‘high activity’ mode. In all cases, activity is either pathologically low or high, or unstable because of the interaction with the environment. As expected, in the low activity mode (Yellow marker), there is very little average activity, and consequently very little “motor” activity; as such the agent remains relatively stationary over time (Fig 3B and 3C). In the high activity phase (Blue Marker), activity levels in the model are consistently high, involving repetitive cycling on-off activity patterns and stereotyped trajectories (e.g., running in a circle). At the phase transition (Green maker), activity levels start relatively low, consistent with rich activity dynamics as reported in many neural simulations at rest (see also S1A/S1B Fig); these rich dynamics result in a relatively rich behavioral trajectory. However, when the agent reaches the boundary wall of the environment where the model receives increased ‘sensory’ input; this leads to increased activity to the model, destabilizing the dynamics and resulting in increased movement and the agent becomes ‘stuck’ (C). This suggests that a model that is tuned to show rich activity dynamics can demonstrate exploratory behavioral dynamics, but that these become destabilized by external feedback into the system in the absence of other stabilizing mechanisms.

### Local homeostasis enables the emergence of rich exploratory behavioral dynamics

To explore how rich behavioral dynamics may emerge from our computational model as a function of local homeostatic mechanisms, we started by exploring the parameter space of key variables controlling local plasticity (see Materials and methods). Using the parameters described above at the optimal regime for the non-homeostatic model (although similar results obtain in other parts of the parameter space) we systematically varied the target rate (i.e., the target for tuning local inhibition) and the learning rate.

**Fig 4:**
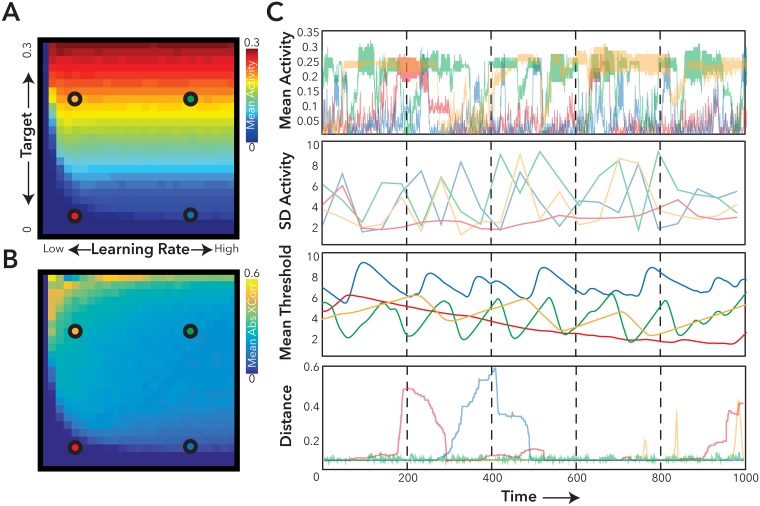
The effect of homeostatic activity on the dynamics and behavior of the agent. Here, we explored the simple homeostatic learning rule applied to the Agent (see Materials and methods). **A-B)** In general, the homeostatic rule could faithfully reproduce the target learning rate, except in the case of very low learning rates. **B)** Application of the homeostatic learning rule was also associated with the emergence of weak-correlation between individual nodes. **C)** Exemplar activity within 4 (Red, Orange, Green and Blue Markers) positions of the learning parameter space are plotted, demonstrating the emergence of correlated changes in both activity and threshold with movement dynamics, and activity.

Consistent with previous results [21], we observed that over time the threshold weights (i.e., the level of local inhibition) adapt so that time-averaged excitatory activation approximates the pre-specified target activity. In Fig 4, we illustrate four different examples of dynamic regimes, exploring both target and learning rate. We note that where the model has a low target rate, and a high rate of learning (Fig 4, blue marker), the model is both able to attain the target value over time, but also in contrast to elsewhere in the parameter space, shows rich activity dynamics (S2 Fig), with the best–fit to a power-law scaling in activity cascades (See Materials and methods). The model also displays non-zero but relatively weak positive correlations between the node activity time-courses which are also consistent with rich asynchronous dynamics (Fig 4B).

**Fig 5:**
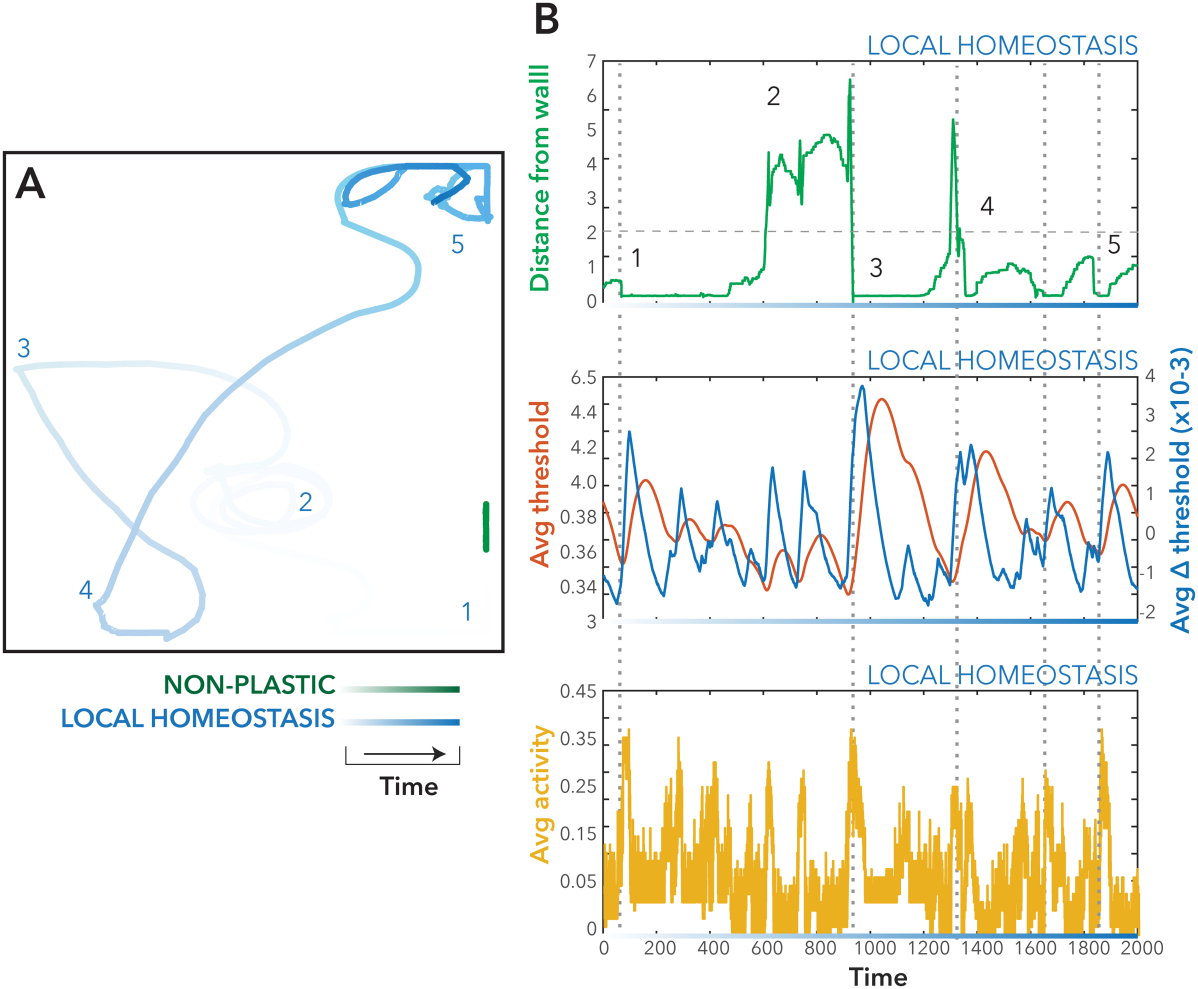
Behavioral dynamics within the non-plastic and local homeostasis forms of the agent model. Models were run for 2000 epochs, and the movement of the model was plotted into real coordinate space **(A)** for the Non-plastic (Green trails), Local Homeostatic (Blue Trails) Model. **B** An example of the emergence of anti-correlations between average activity, and distance from the wall of the agent in comparison with the change in threshold over time. Dotted lines demonstrate points in time when the agent collided with the bounding wall.

To understand how the local homeostatic plasticity stabilizes activity and how this relates to movement dynamics, we selected parameters for the local homeostatic model in a low-target activity, high-learning rate regime (Fig 4, Blue Marker), and contrasted the non-homeostatic and local homeostatic models (Fig 5). Over time the local homeostatic model moves into a regime with generally higher levels of movement (i.e., left/right rotation and/or forward motion) (Fig 4C), although there is considerable variability (i.e., the mean level of movement and activity varies considerably over time). Example trajectories for both the static and local-feedback model (over 2000 epochs) are presented in (Fig 5). The entropy of the movement dynamics (measured using the entropy of the movement and turn motor signals generated by the model) was significantly higher in the local homeostatic model compared to the static model, (t_58_ = 2.68 p<0.05). Moreover, the fractal dimension of the movement (i.e., the fractal dimension of the image resulting from the trajectory) was significantly increased in the local plasticity model compared to the static model (t_58_ = 11.76, p<0.001)—See Also S3 Fig.

Non-stationary dynamics in both the simulated neural dynamics and the behavior of the agent reflect a feedback loop arising from how the agent interacts with the environment. The level of sensory input:

1. Alters the level of simulated activity, which;
2. Alters the level of motor output,
3. Manifests in agent movement, which in turn;
4. Alters subsequent sensory input.

This brain-environment interaction can be observed by the significant anti-correlation between distance from the wall, and activity of the static model (t_26_ = -3.93, p< 0.04). We can also observe how the local-homeostatic mechanism allows the model to escape from brain-environment feedback loops, by increasing or decreasing local inhibition to better approximate the target activity rate. There was a significant decrease in this anti-correlation within the local homeostatic model compared to the static model (t_58_ = -2.78, p<0.01). We see that for the local homeostatic model, there was a significant anti-correlation between the mean threshold and both the distance from the wall (t_58_ = -9.63, p<0.001) and mean threshold and mean activity (t_58_ = -12.76, p<0.001). This shows how the coupling between activity and distance from the wall was decreased in the local homeostatic model compared to the static model, as the model retunes the local thresholds to compensate for increased activity (driven by sensory input) and re-establish richer movement dynamics.

### Macroscopic balancing enhances functional and behavioral dynamics

We observed that the local-homeostatic mechanism compensates for the brain-environment feedback, by constantly readjusting weights to compensate for the non-stationary environment. By adding a complementary balancing mechanism that aims to keep activity levels approximately constant across regions with the local homeostatic mechanism (aiming to balance activity across time), a more stable solution can be arrived at.

**Fig 6:**
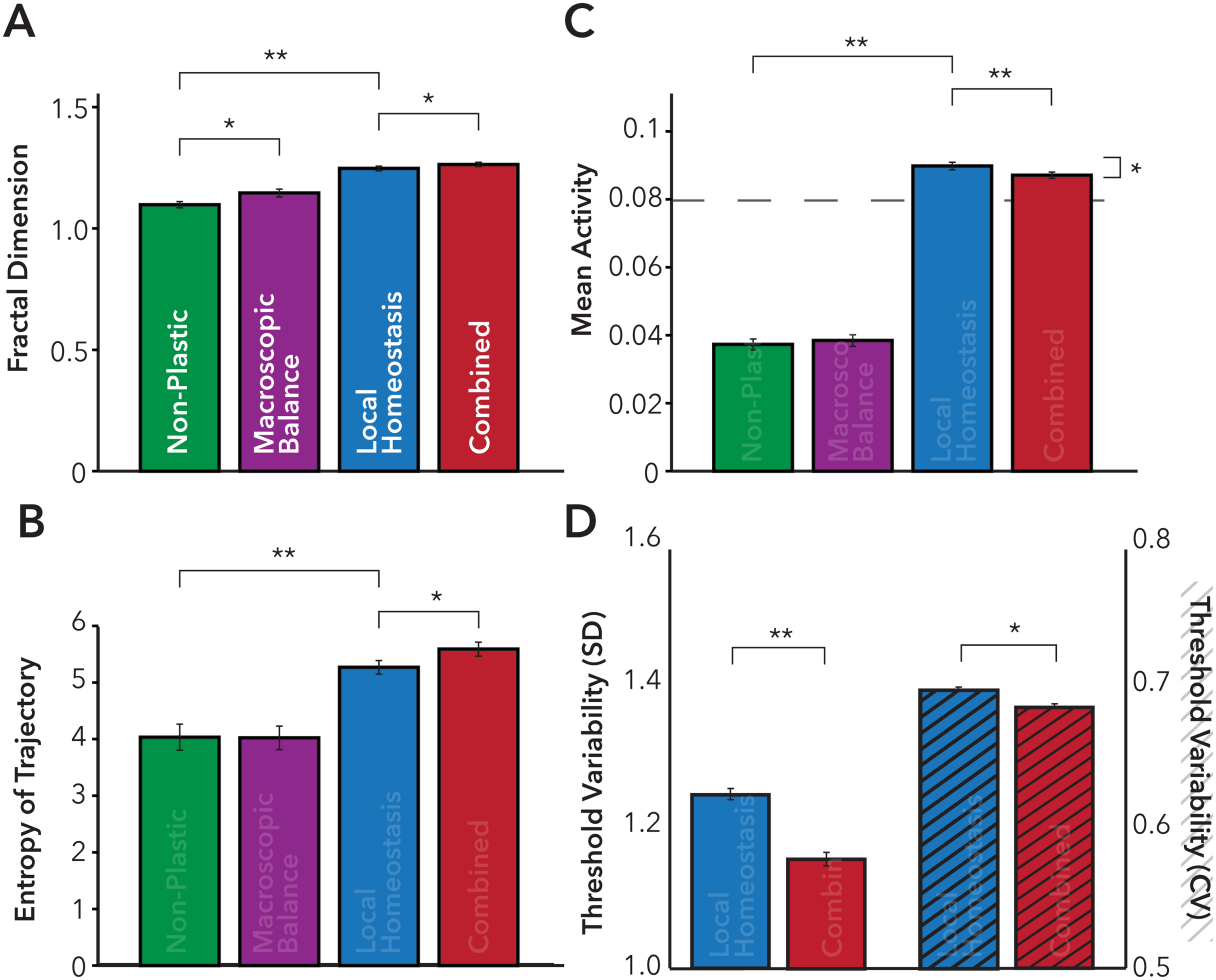
Behavioral and functional dynamics of the computational model. Plots of Fractal Dimension **(A)**, and Entropy of the movement dynamics **(B)** for each version of the computational model. **C)** Mean activity over 2000 epochs of the computational model—Dashed line represents the overall target rate of the homeostatic tuning function selected for the Homeostatic and Combined local and macroscopic models. **D)** Exploration of the threshold variability for the Local Homeostatic (Blue) and Combined local and macroscopic (Red) Models. (n = 30 repeats of the model ± 1 SEM, ** = p<0.001, * = p<0.05).

We compared the local-homeostatic, macroscopic and the combined (local and macroscopic homeostatic) models (see Fig 1) on a range of measures assessing the model’s activity and movement dynamics (Fig 6). We noted that there was a significant difference in mean activity across the network in the combined local and macroscopic model, compared to the local-homeostatic model (t_58_ = -2.89 p< 0.01); the model with macroscopic balance alone, was not significantly different in terms of mean from the non-plastic model (t_58_ = -0.49 p = 0.63). In addition, variability of the model measured using the mean standard deviation of activity across network nodes (t_58_ = -3.07p< 0.01) was significantly decreased in the combined model. This suggests a small decrease in mean activity in the macroscopically balanced model compared to the local-homeostasis model, with activity significantly closer to the homeostatic target function (t_58_ = -2.87, p<0.01) for the combined model (Fig 6C).

More importantly, in the combined model, threshold weight changes are significantly less variable than for the local homeostatic model when considering both the standard deviation and the coefficient of variation of the mean threshold over time (t_58_ = -6.94, p<0.001) and (t_58_ = -2.60, p<0.05) respectively (Fig 6B). These results suggest that the combined model arrived at a more stable behavioral interaction than local–homeostasis alone, requiring less local weight change in response to persistently elevated or reduced activity. In addition, the relationship between the distance from the wall and mean activity is significantly less for the macroscopically-balanced model (t_58_ = 2.46, p<0.05) than the local homeostatic model alone—suggesting the feedback loop between brain/environment is less influential.

The more stable activity and weight change of the combined model manifests itself in a richer behavior, and more complete exploration of the environment. When observing the movement of the agent we see that (Fig 5A), the path of the agent has a higher fractal dimension (t_58_ = 2.2, p<0.05) for the combined model and significantly higher entropy for the plotted trajectories (Fig 6A/6B). The fractal dimension for the macroscopic balance alone model however, was significantly higher than the non-plastic model (t_58_ = -2.35, p<0.05). These results suggest that the agent has a more complex pattern of activity, that emerges from a multi-scale balancing system and covers more of the environment because behavior is less determined by brain-environment interactions alone (S3 Fig).

## Discussion

This model is unequivocally not intended to be a detailed model of all aspects of real embodied cognition or of actual neural and sensorimotor systems; instead, in both regards, it is highly simplified. We acknowledge that there have been many arbitrary design choices, and do not intend this to be a definite presentation of how to model brain/environment/behavior interactions. Such interactions are likely to be far more complex, possibly non-stationary, and will depend on the complexity both of the neural system, but also the complexity of the motor and sensory systems. Instead, the example we present here is a useful toy example; the simplification allows us to consider the interactions between macroscopic brain networks [23], neural dynamics and the environment to better understand possible functional roles of homeostatic systems in the brain.

The presence of a local homeostatic plasticity mechanism that tunes the threshold at each node to balance excitatory input from connected nodes ensures that the agent does not stay trapped in either state for long. As the threshold for activity (varying depending on the local homeostatic mechanism) at individual nodes increases (in the high activity state) or decreases (in the low activity state), the average activity level adapts to the target level. This results in the agent escaping the ‘trap’, with resulting activity levels closer to the target level and, consequently, more stable simulated neural and movement dynamics.

The model without local homeostasis is unable to cope with the sensory/motor feedback system. Local thresholds can be chosen to allow interesting dynamics (i.e., variable movements/neural activity); however, these must be chosen to either allow rich dynamics in the presence of sensory input (i.e., with higher local inhibition) or dynamics in the absence of sensory input (i.e., with lower local inhibition). Therefore, over time the agent will tend to either: a) remain approximately stationary in a low-sensory area with local thresholds too great to allow much exploration (i.e., near stationary) or b) initially move freely, but rapidly become trapped in a high-sensory area (e.g., a corner or wall).

The model suggests that modeling spontaneous dynamics at rest (e.g., [24–26]) or with a simple task such as encoding a sensory stimulus (e.g., [15–16]) is different to modeling sensori-motor interactions with an environment; further, the existence of closed-loop feedback made the roles of homeostatic mechanisms more important and obvious. In our case, we observed that without the local homeostatic plasticity, the agent in the environment would become trapped in either a stationary state (with high levels of local inhibition) or would be in a permanent state of motion (with too little local inhibition). Instead, we observe that plasticity is a constant feature of the system. Initially, there are large changes in local thresholds across time points, as the model approximately balances average incoming excitation at each node. As time progresses, however, the weight changes become smaller, but never drop completely to zero.

While the model with local homeostasis can compensate for this sensory-motor interaction, the addition of an explicit macroscopic balancing system across space, alongside the local homeostatic learning rule (that balances activity across time), further facilitates stable simulated neural dynamics and behavioral trajectories through the environment. This occurs because the macroscopic system balances alterations in external input to the model so that the number of activated units (sensory nodes or task negative nodes) remains constant irrespective of interactions with the environment. The simple system we implemented, modeled on patterns of task negative deactivation from the fMRI/PET literature (e.g., [11]) counteracted the destabilizing effects from the changing amount of sensory input that the model receives in different locations in the environment. Without the macroscopic system, the overall level of activity within the model is more dependent on the level of sensory input (i.e., “touching” and “seeing” the wall). This makes the task of the local homeostatic plasticity mechanism harder, since exogenous input to the system varies considerably. Instead, the task negative system simply balances the level of exogenous activity to a constant amount, such that task negative input decreases as sensory input increases and vice versa. This means that the environment/brain feedback loop does not change the overall level of incoming activity to the model, therefore facilitating the local homeostatic plasticity to find a more stable solution, i.e., one that requires the smallest weight changes to approximate the target activation rate. Further, what we observe are different balancing systems operating at different spatial and temporal scales and with different specific mechanisms. This is consistent with the proposed description of normalization found in many neural systems [27]. Indeed, in our previous work, we described the computational complexity of behavior that emerges naturally out of systems that account for spatial and temporal interactions across a range of scales [14]. Such an architecture provides a canonical computation across scales and implementations, and results in improved neural coding efficiency and sensitivity.

From a traditional cognitive neuroscience perspective, this way of thinking about task negative systems may sit somewhat uncomfortably. What we have been describing as task negative may provide a partial functional explanation for the default mode network. The default mode network is a well-characterized, frequently observed and relatively poorly understood macroscopic brain network located in areas of the brain not associated with sensorimotor activity; the default mode network has been observed across ontogeny [28], phylogeny [29], and found across different cognitive and sensorimotor tasks [30] and implicated in many disorders [31]. According to our findings, the default mode network can be thought of as acting as a counterweight, or as an endogenous generator of neural activity that allows the neural system to remain relatively stable in an inherently unstable world. One analogy could be to the vascular system of warm-blooded animals, which attempts to maintain a constant body temperature, irrespective of the temperature outside, to maintain a stable environment for chemical reactions to take place, ultimately allowing more flexible behavior. We note that the proposed balancing functional role for task negative brain networks does not preclude more traditional cognitive roles ascribed to them, such as mentation. We hypothesize that task negative systems could have initially evolved to perform some basic neural function, such as balancing incoming sensory activity, and eventually been adaptively repurposed over evolution to perform more specific cognitive functions that occur when external input is not present, as such, task negative systems reflect a macroscopic-scale spatial ‘mirror’ of local temporal homoeostatic normalization rules, demonstrating a multi-scale architecture in the brain for normalization processes [14].

Whilst our model only considers a relatively simple link between dynamics and functional behavior, our observations are consistent with our previous work exploring stability of neural dynamics in the brain and more complex sustained tasks [15]. From our model that suggests that complexity and flexibility of behavior is associated with efficient task-positive/negative interactions, we predict that disruption to task-negative regions of the brain such as the default mode network would be associated with disruption to sensory or motor processing; however, these disruptions could involve both increases and decreases in neural activity following sensory input; may take time before they manifest themselves and may be associated with less flexible behavior. This prediction is consistent with previous neuroimaging and behavioral work in Traumatic Brain Injury (TBI) [32–35]. Exploring the relationship between task-positive and task-negative balance in more complex tasks and in pathologies known to affect local E/I balance (e.g., the E/I disruption model of Schizophrenia [36]) using this computational framework compared to empirical studies is the subject of ongoing work.

Following this explanation of the task-negative balancing systems in general and the default mode network more specifically, we see that task-negative systems may not strictly be “necessary” for accomplishing any task. As such, lesioning task negative regions is unlikely to disturb any associated function entirely, and as such task negative systems may appear to be epiphenomenal. However, just as a sailing boat does not require a keel to move (the keel counterbalances the forces on the sail, facilitating stability and allowing a wider range of movement and greater speed), the brain may have a greater range of neural state and potentially be more controllable, when it is properly counterbalanced. It might only be over longer time periods when initially adapting to a novel environment or across development that damage to task negative systems becomes particularly disabling, failing to facilitate other adaptive systems as efficiently.

In the current results, we observed only a small (but significant) enhancement in behavioral and neural dynamics for the combined macroscopic and local-homeostatic models over the local-homeostasis only model, and no difference between the macroscopic homeostasis model and the static difference. This smaller effect suggests that the local-homeostasis is more important for promoting rich spontaneous dynamics; however, it does not mean that the macroscopic homeostasis is irrelevant. In the combined mode, macroscopic balancing was added to a local-homeostasis model that was tuned to perform optimally, unlike the macroscopic homeostasis model; this showed that the macroscopic mechanism could augment the local homeostatic model. Future work will investigate potential mechanisms that may underlie and tune the macroscopic spatial homeostasis; these have the potential to greatly increase both the independent and synergistic roles of the macroscopic homeostasis mechanism and make testable predictions about the optimal spatial organization and connectivity of task positive and negative networks.

The location of the sensory input systems, motor output systems and task-negative nodes were chosen relatively arbitrarily. This is because the coarse resolution of the parcellation means that assigning sensory or motor labels to nodes is inherently very approximate. As such, we do not wish to draw parallels with specific brain regions or networks (e.g., from the functional imaging literature), however, understanding the true relationship between task positive and task negative nodes is the focus of our continued research using computational modelling approaches like that described here.

Finally, to achieve a relatively stable solution with rich spontaneous dynamics and interactions with the environment, the system may have to encode (in the local inhibitory weights) information about the world, and the agent’s movement in it. Given the relative simplicity of the environment in the current simulation, the presence of local thresholds is adequate to facilitate a relatively stable solution. However, as the environment (and sensory input systems) becomes more complex, it will be necessary to use more sophisticated models with more flexibility. If the repertoire of brain states is to be more fully explored in the face of this increasing complexity, then it will be necessary to capture more information about the environment/sensory systems. This leaves open questions about the roles of other types of learning (e.g., longer-distance excitatory and reinforcement learning) and their roles in supporting the system staying in a rich dynamical regime, in a complex environment, with complex sensorimotor systems and with more cognitive control mechanisms.

## Methods

### Empirical structural connectivity

Simulated activity patterns were generated from a computational model constrained by empirical measures of white-matter structural connectivity between 66 cortical regions of the human brain, defined by diffusion tensor imaging (DTI) [18]. This structural network has been used in a range of previous computational models to demonstrate emergent properties of resting state functional connectivity [15,20–21,25,33]. A full methodology, describing the generation of the connectivity matrix 〈*C*〉 is available in [18]. In brief: measures of length and strength of stream-line based connectivity were estimated using Deterministic tractography of DSI datasets (TR = 4.2s, TE = 89s, 129 gradient directions max b-value 9000s/mm^2^) of the brain in 5 healthy control subjects. A high-dimensional ROI based connectivity approach was projected though the 66 regions of the Desikan-Killianey atlas (FreeSurfer http://surfer.nmr.mgh.harvard.edu/), such that *C*_*i*.*j*_ is the number of streamlines connecting nodes *i* and *j*.

### Computational model

#### Neural dynamics

To simulate brain activity, we defined a simple method based on the Greenberg-Hastings model, which has been shown in previous work to approximate patterns of empirical functional connectivity [19]. The local dynamics of the model were defined such that at each time point, *t*, each node, *i*, in the model can be in one of three states, *S*_*i*,*t*_: excitatory (E), quiescent (Q), or refractory (R). Nodes changed state according the following simple probabilities: *p*_*i*_ (*E* → *R*) = 1; *p*_*i*_ (*R* → *Q*) = 1; *p*_*i*_ (*Q* → *E*) = 10^−1^. At the network level, dynamics at each node were also affected by interactions with their neighbors as nodes would also change from Q->E if the summed input from the set of *n* of each connected node, *j*, was greater than a specified local threshold value: $\Sigma_{j=1}^{n}C_{i,j}\;S_{j,t-1}>T_{i}$. The strength of the activation threshold, *T*_*i*_, could be tuned to separately at each node (see below). *S*_*i*,*t*_ was binarized so that E was coded as 1, R or Q as 0.

#### Homeostatic plasticity

For simulations that included plasticity at the local level, we used a local homeostatic plasticity mechanism as follows: we allowed the activation threshold to vary by a small amount based on the activity in each node at the previous time-step, according to the following rule; similar (but simplified) to that introduced in [3] and used in [21]: *δt*_*i*_ = *α*(*S*_*i*,*t*_ − *ρ*). Where *ρ* is a mean target activation level and *α* is a learning rate. Thus, in the case that the activity of *i* is 1, and *ρ <* 1 the threshold will increase, whereas, otherwise the threshold decreases. Thus, the time-averaged activity of *S*_*i*_, will approximate *ρ*.

#### Avalanche dynamics

To assess dynamics consistent with criticality, we used a similar approach to that described in previous work, to explore the presence of power-law scaled ‘avalanche’ dynamics within the activity of the model [21,37]. In brief; The classical definition of neuronal avalanches describes periods of bursting activity within a neural system, bounded by periods of quiescence. Discretized activity (related to the active ‘on’ state) across the entire system was (optionally) re-sampled temporally into bins of Δ*t*. Avalanches (or cascades) were defined as a continuous sequence of time-bins within which an event occurred somewhere within the system, bounded by time-bins where network activity was silent. The size of the cascade (*K*) was defined as the number of individual events that occur within each avalanche. It has been repeatedly observed using this approach that the probability distribution of the cascade size *P* (*K*) within a critical system is scale free, distributed as power law where *P*(*n*)~*n*^−3/2^ [37–39]. To assess the ‘goodness of fit’ of power-law distributed probability distributions, to a putative reference distribution, we used a standard least-squares fitting approach [38]. In addition, we explored a ‘distance’ measure from a perfect power-law as described in previous work by ourselves and others [21,39–41].

#### Environmental Embedding (Fig 1C–1E)

To embed the computational model into a virtual environment; the motor activity (movement) of the agent was defined by two commands; Turn (*h*) in radians per update step and Move (*v*) which moved the agent forward v world units. The activity within these two parameters at each time-step was determined by the simulated neural activity at four nodes (two rotate and two advance nodes) of the computational model. We chose these nodes to be bilaterally symmetrical such that they approximately correspond to motor related regions in the brain (*n*.*b*., *we do not aim to make claims that this anatomical correspondence is specific*, *correct or that the results are dependent on the exact location of nodes*, *a further exploration of the topological relationship between task positive and task negative nodes is the subject of our ongoing work*). If, in the normal dynamic functioning of the computational model, a ‘rotate’ node was active, the agent would attempt to rotate ~30° in that direction. If both nodes were active, then the effect would cancel this out effectively by insisting that the agent rotate equally in opposing directions. If a single forward node was active, the ‘forward’ motion of the agent would increase by one half a unit in the arbitrary world space, if both forward nodes were active, the unit would accelerate by 2 units of world space (Fig 1C). In addition, we added some temporal smoothing across time for activity within the move such that the move command described was 7/8 of the activity of the relevant assigned node, and 1/8 of the activity of the previous time step. (The amount of this smoothing and the values of how nodes translated into movement were chosen semi-arbitrarily, to produce agent motion that appeared superficially plausible, i.e., neither very fast or slow).

Sensory information (‘visual’ perception) from the environment was integrated into the computational model using two horizontal ray-traces emanating from each “eye” of the agent and offset by ±10° from the vertical. A distance threshold was defined, such that if an object (i.e., the wall, in this simple environment) was less <2 units of world space then a specific node (“near visual”) of the model was set to the E state, if an object was detected between 2 and 10 world units away then the “far visual” node was set to excitatory (Fig 1D). In addition to “visual” input, we also defined a rudimentary “somatosensory” input, whereby if agent collided with any other object in the environment then specific “somatosensory” nodes for collisions on either of the Left or Right side of the agent were set to the E state (Fig 1E).

#### Task-negative nodes (Macroscopic Homeostasis balancing)

To explore the effect of balance between sensory task positive (TP) and task negative (TN) networks in models with macroscopic balance we defined a collection of TN nodes that were anti-correlated with the TP (“visual” and “somatosensory”) nodes described above. These TN nodes were defined as two (bilateral) pairs of task negative nodes approximately corresponding to regions that consistently show relative deactivation across many empirical fMRI tasks were chosen (*although*, *this was still a relatively arbitrary decision and we do not wish to make any claims based on anatomical precision*). These nodes were set to the E state if TN nodes (i.e., the “visual”, or “somatosensory” nodes were in the Q or R states, and Q, when the TN nodes were activated, such that activation of the TP nodes were associated with an anti-correlated deactivation of the TN nodes.

#### Supplementary materials

The code/implementation (in Unity) and compiled versions of the model are available at https://github.com/c3nl-neuraldynamics/Avatar/releases.

## Supporting information

S1 Fig Power-law scaling in avalanche dynamics associated with phase-transition in the simulated neural dynamics. Top: cascade Size (i.e. the number of regions of the model involved in a putative neural avalanche) Bottom: cascade Length (i.e. the distribution of the lengths of each putative neural avalanche). Here we present Kappa (relative to a perfect power-law with an exponent of -1.5) and the negative log-likelihood of a perfect power-law fit.
(EPS)

S2 Fig Power-law scaling in avalanche dynamics associated with local homeostatic plasticity in neural dynamics. Cascade Size (i.e. the number of regions of the model involved in a putative neural avalanche). Here we present the negative log-likelihood of a perfect power-law fit of neural dynamics. Red, Orange, Green and Blue Markers represent the same regions of this parameter exploration as those presented in Fig 3.
(EPS)

S3 Fig Behavioral dynamics within the non-plastic and local homeostasis forms of the agent model. Models were run for 2000 epochs, and the movement of the model was plotted into real coordinate space **(A)** for the Non-plastic (Green trails), Local Homeostatic (Blue Trails) and Combined local and macroscopic (Red Trails) Model.
(TIF)
