# Supplementary figures and images for: From homeostasis to behavior: Balanced activity in an exploration of embodied dynamic environmental-neural interaction
Source: PLoS Comput Biol. 2017 Aug 24;13(8):e1005721. doi: 10.1371/journal.pcbi.1005721 (PMC5587328; doi:10.1371/journal.pcbi.1005721)

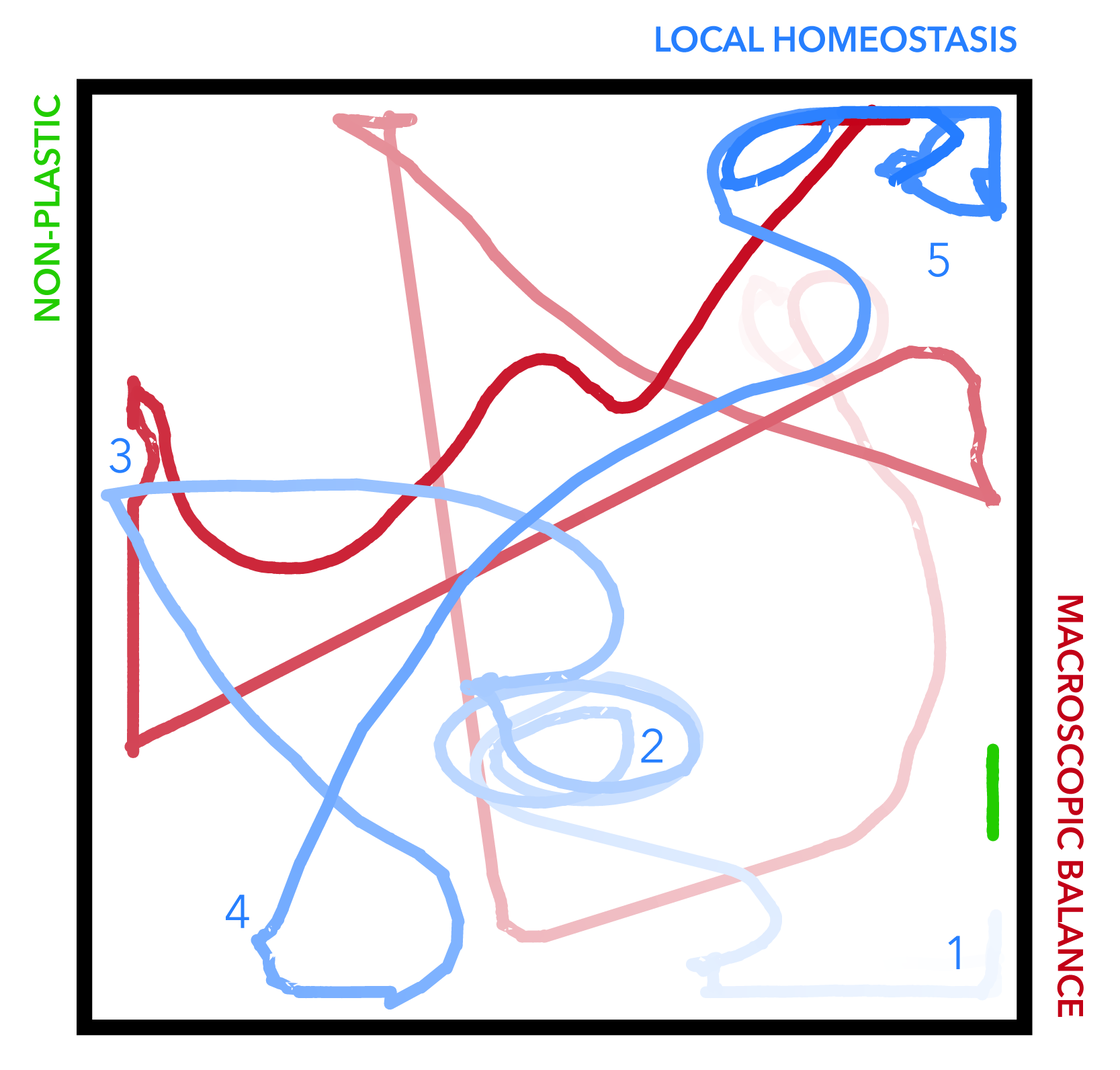

Supplement: S3 Fig — Models were run for 2000 epochs, and the movement of the model was plotted into real coordinate space (A) for the Non-plastic (Green trails), Local Homeostatic (Blue Trails) and Combined local and macroscopic (Red Trails) Model. (TIF) [file pcbi.1005721.s003.tif]
